# Dexamethasone Suppressed LPS-Induced Matrix Metalloproteinase and Its Effect on Endothelial Glycocalyx Shedding

**DOI:** 10.1155/2015/912726

**Published:** 2015-06-23

**Authors:** Na Cui, Hao Wang, Yun Long, Longxiang Su, Dawei Liu

**Affiliations:** Department of Critical Care Medicine, Peking Union Medical College Hospital, Beijing 100730, China

## Abstract

The aim of this study is to determine the mechanism of sepsis-induced vascular hyperpermeability and the beneficial effect of glucocorticoid in protecting vascular endothelium. Male Sprague-Dawley rats were given either a bolus intraperitoneal injection of a nonlethal dose of LPS (*Escherichia coli* 055:B5, 10 mg/kg, Sigma) or vehicle (pyrogen-free water). Animals of treatment groups were also given either dexamethasone (4 mg/kg, 30 min prior to LPS injection) or the matrix metalloproteinases (MMPs) inhibitor doxycycline (4 mg/kg, 30 min after LPS injection). Both activities and protein levels of MMP-2 (*p* < 0.001) and MMP-9 (*p* < 0.001) were significantly upregulated in aortic homogenates from LPS-treated rats, associated with decreased ZO-1 (*p* < 0.001) and syndecan-1 (*p* = 0.011) protein contents. Both dexamethasone and doxycycline could significantly inhibit MMPs activity and reserve the expressions of ZO-1 and syndecan-1. The inhibition of MMPs by dexamethasone was significantly lower than that by doxycycline, while the rescue of syndecan-1 expression from LPS-induced endotoxemic rat thoracic aorta was significantly higher in the dexamethasone-treated compared to the doxycycline-treated (*p* = 0.03). In conclusion, activation of MMPs plays important role in regulating ZO-1 and syndecan-1 protein levels in LPS mediated endothelial perturbation. Both dexamethasone and doxycycline inhibit activation of MMPs that may contribute to the rescue of ZO-1 and syndecan-1 expression.

## 1. Introduction

Severe infection or sepsis is caused by a variety of kinds of toxins, which are released by bacteria including nitric oxide, and many proinflammatory cytokines or chemokines [1]. Accumulation of these proinflammatory cytokines and cascade of inflammatory reaction following the severe infection could ultimately cause abnormal vascular hyperpermeability and abnormal activation of coagulation pathways. This could result in abnormal microcirculation and obstruction of perfusion [2]. In addition, proteinases including matrix metalloproteinases are upregulated and activated in response to stimulation of proinflammatory cytokines, which leads to degradation of extracellular matrix, and breaking down tight junctions and other molecules connecting cell-cell structures [3, 4]. Degradation of extracellular matrix and breaking of tight junctions between endothelial cells contribute to the further development of abnormal leakage, edema, and multiple organ failure [5, 6]. Therefore, microcirculation dysfunction is the core mechanism of severe infection. How to maintain the integrity of the vascular endothelial cell structure and function and reduce excessive activation of endothelial cells and the damage has become a hot topic in physiological and pathological study in the field of sepsis [7].

Vascular endothelial cells play an important role in maintaining homeostasis. Under normal physiological situation, endothelial cell surface is covered with a layer of glycocalyx proteins, which mainly consisted of syndecan and glypican families. Glycocalyx, cellular tight junction, and other cell-cell connecting proteins play an important role in maintaining vascular homeostasis [8]. These structural barriers or junctions are damaged when a severe infection or sepsis occurs. In this regard, endotoxin together with accumulated proinflammatory cytokines could initiate a cascade of inflammatory reaction, activate endogenous proteinases including matrix metalloproteinases, and, thus, lead to impaired mechanotransduction with changes in fluid shear stress, activation of coagulation pathways, adhesion of leukocytes and platelets to the endothelial cells surface, and leakage of fluid and plasma proteins into the interstitial tissue [9]. Therefore, protection of glycocalyx and endothelial cell tight junctions seems to be a promising therapeutic goal in the scenario of severe infection or sepsis.

Recent studies indicated that MMPs play an important role in the development of ischemia-perfusion injury through damaging glycocalyx layer of endothelial cell surface [10]. Doxycycline is known to inhibit a variety of kinds of MMPs and thus widely used to investigate role of MMPs in many clinical scenarios including cancer metastasis and sepsis [11–13]. Steroid has been known to inhibit expression and activation of MMPs in cell culture as well as animal models [14]. In the current study, therefore, using an animal model of endotoxemia, role of MMP-2 and MMP-9 in mediating LPS-induced degradation of ZO-1 and syndecan-1 was investigated.

## 2. Materials and Methods

### 2.1. Animals

This study was approved by the Institutional Animal Care Committee from Chinese Academy of Medical Sciences and all procedures were conformed to the “Guidelines for the Care and Use of Laboratory Animals,” as published by the National Academy Press (NIH publication number 85-23, revised 1985). A total of 40 adult and male Sprague-Dawley (SD) rats (250–350 g) were obtained from the Animal Research Institute, Chinese Academy of Medical Sciences (Beijing, China). They were divided into five groups (8 in each group): Control group, LPS group, dexamethasone group, LPS + dexamethasone group, and LPS + doxycycline group. Endotoxin (*Escherichia coli* 055:B5, Sigma, USA), 10 mg/kg, was intraperitoneally injected. Dexamethasone (4 mg/kg) was injected 30 minutes before LPS injection in the groups of dexamethasone alone or LPS + dexamethasone. Doxycycline (4 mg/kg) was injected 30 minutes after LPS injection. All animals were sacrificed humanely by pentobarbital sodium overdose (100 mg/kg ip) after 6 hours of endotoxin injection [15, 16]. Blood samples and thoracic aortas were harvested for the studies as designed (Supplemental Figure 1 in Supplementary Material available online at http://dx.doi.org/10.1155/2015/912726).

### 2.2. Immunohistochemistry

Aortic tissues were harvested and fixed with 2.5% (v/v) glutaraldehyde-polyoxymethylene solution immediately after rat was in euthanasia. The tissue samples were dehydrated and embedded in paraffin wax. Serial paraffin sections (4 *μ*m) were obtained and immersed in three consecutive washings in xylene for 5 min each to remove paraffin and then hydrated with five consecutive washings with alcohol in descending order, 100, 95, 80, 70, and 50%, and deionized water, respectively. Citrate buffer was used for antigen retrieval under high temperature and pressure conditions. H_2_O_2_ was used to reduce nonspecific antigen deposition. Sections were immune stained with anti-MMP-2 oranti-MMP-9 antibody (1 : 300; AB-38917, Abcam, Cambridge, UK; AB-119906, Abcam, Cambridge, UK) by incubating at 4°C overnight. Next day, a biotin-labeled secondary antibody was applied. Diaminobenzidine and hematoxylin were used to visualize the staining. Three fields per section and three sections per rat were analyzed under microscope.

### 2.3. Gelatin Zymography

Protein was extracted from the rat aortic tissue homogenates. After determination of protein centration, the protein from each group was loaded with nonreducing loading buffer for SDS-PAGE. Gelatin is embedded in the resolving gel during preparation of the acrylamide gel. Following electrophoresis, the SDS is removed by 2 times washing with 2.5% Triton X-100, followed by incubation in digestion buffer overnight at 37°C. The zymogram is subsequently stained with Coomassie Brilliant Blue followed by destaining, and areas of digestion appear as clear bands against a darkly stained background where the substrate has been degraded by the MMPs. Gel pro 4.0 (Media Cybernetics, Silver Spring, MD, USA) was used to analyze gel optical density of each sample.

### 2.4. Immunoblotting

Total protein from aortic specimens was extracted using RIPA Lysis Buffer (Applygen Gene Technology Corp., Beijing, China), and the amount of protein was measured using the bicinchoninic acid method. Immunoblotting was performed using anti-MMP-2 (1 : 300; AB-38917, Abcam, Cambridge, UK), anti-MMP-9 (1 : 300; AB-119906, Abcam, Cambridge, UK), and oranti-ZO-1 antibodies (1 : 200; Santa Cruz Biotechnology, Inc.). Horseradish peroxidase-labeled goat anti-rabbit IgG was applied as secondary antibody (Applygen Gene Technology Corp., Beijing, China). Detection was performed using the ECL kit (ZSGB-Bio, China), and quantification was performed using the Gel pro 4.0 (Media Cybernetics, Silver Spring, MD, USA).

### 2.5. ELISA Assay

Aortic homogenates were purified by centrifugation and used for syndecan-1 quantification by ELISA using a commercially available kit (SEB966Ra, USCN Life Science Inc., China) following the manufacturer's instruction.

### 2.6. Statistical Analysis

All data are expressed as mean ± SD. Statistical comparison among five groups was evaluated by one-way analysis of variance (ANOVA) followed by LSD or SNK-q protected least significant difference test between any two groups. SPSS 16.0 software was used for all analyses. Values of *p* < 0.05 were considered statistically significant.

## 3. Results

### 3.1. Observation on the Animals following Endotoxin Injection

After injection of LPS, behavioral change was closely monitored in each group of animals. After 45 minutes of LPS injection, the animals with LPS injection gradually manifested sleepy, less active, slow reaction, dry and grizzled hair, tachypnea, chills, and cyanosis, while LPS + dexamethasone and LPS + doxycycline group appeared to be transient apathetic with less movement after LPS administration and recovered to become active with good appetite after 2 hours of injection. At 6-hour time point, blood gas analysis indicated that pH, lactate, base excess, PaCO_2_, and SaO_2_ were significantly altered in the group of LPS injection only compared to the groups of Control, LPS + dexamethasone, or LPS + doxycycline (Table 1).

### 3.2. MMP-2 and MMP-9 Expression in the Aortic Endothelial Cells

**Figure 1:**
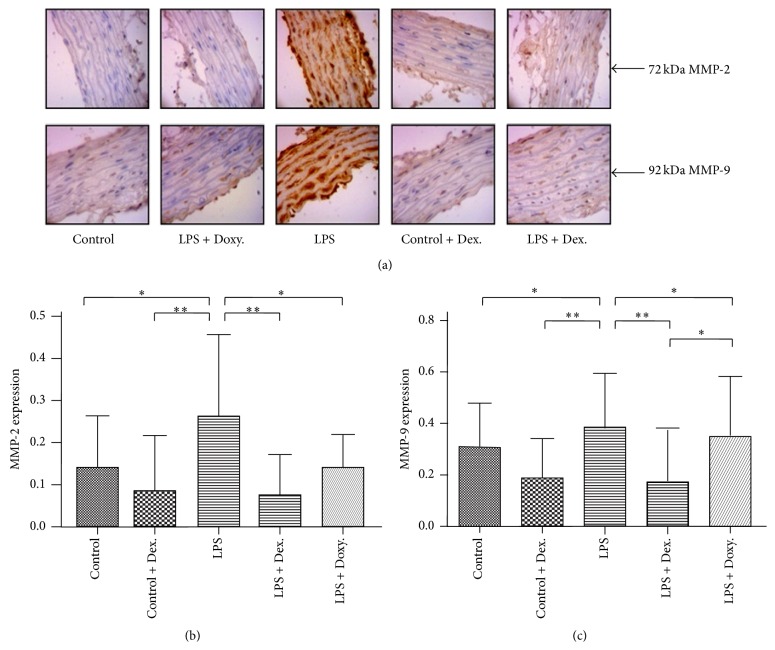
Immunohistochemistry of MMP-2 or MMP-9 expression. (a) As described in the method, expression of MMP-2 (72-kDa) and MMP-9 (92-kDa) in cross sections of thoracic aorta from each group was determined by immunohistochemistry. Data presented is one representative of 8 replicates in each group. (b and c) Average of 8 biological replicated samples from each group (mean ± SD). ^*∗*^ *p* < 0.05; ^*∗∗*^ *p* < 0.001.

Expression of MMP-2 and MMP-9 in the aortic tissues was determined by immunohistochemistry. As shown in Figure 1, under “Control” condition, aortic endothelial cells do not express MMP-2 or MMP-9. Dexamethasone alone had no effect. In contrast, LPS significantly stimulated MMP-2 and MMP-9 expression in the aortic endothelial cells as evidenced by brown staining of the cells. In the presence of dexamethasone or doxycycline, however, LPS-induced expression of MMP-2 and MMP-9 was completely inhibited (*p* < 0.05). Of note, LPS-induced MMP-9 expression was further inhibited by dexamethasone compared to that by doxycycline (Figure 1(c), *p* < 0.05).

### 3.3. MMP-2 and MMP-9 Expression and Activation

**Figure 2:**
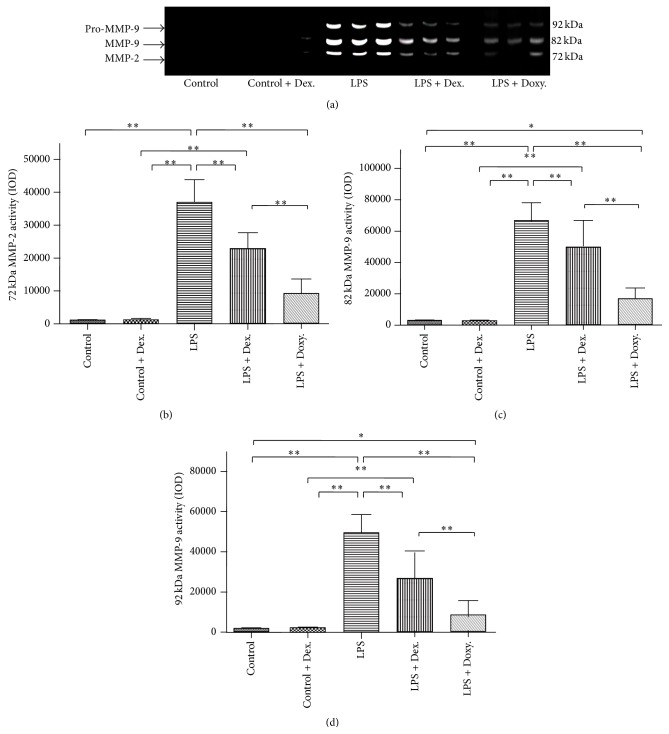
Gelatin zymography. Proteins of the aortic homogenate from each group were used for zymographic assay as described in the method. (a) One representative image data of zymogram. 72-kDa: latent MMP-2; 82-kDa: active MMP-9; and 92-kDa: latent MMP-9. (b, c, and d) Densitometric analysis of 8 biological replicates of latent MMP-2 (72-kDa), active MMP-9 (82-kDa), and latent MMP-9 (92-kDa), respectively. ^*∗*^ *p* < 0.05; ^*∗∗*^ *p* < 0.001.

**Figure 3:**
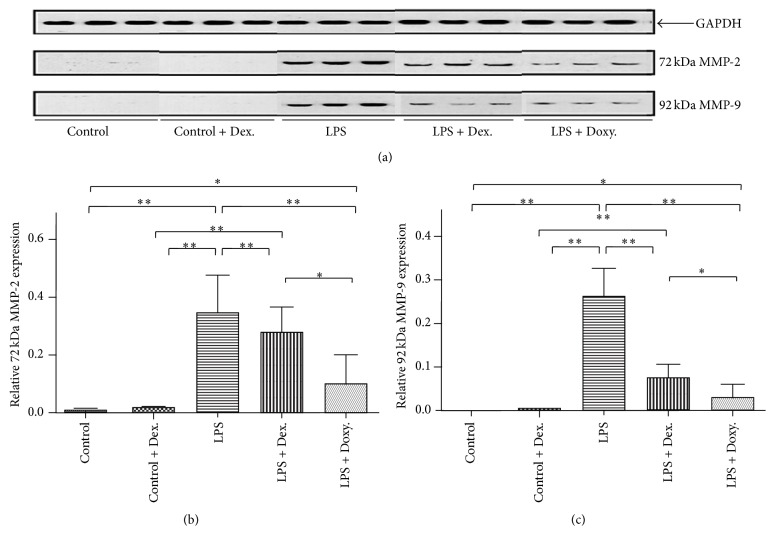
Immunoblotting of MMP-2 and MMP-9. Proteins were extracted from the aortic tissues of indicated treatment groups and immunoblotted for MMP-2 and MMP-9 as described in the method. (a) Immunoblotting image of MMP-2 and MMP-9 among different groups. GAPDH was used as loading control. Latent MMP-2 (72 kDa) and MMP-9 (92-kDa) were detected as indicated. (b and c) Densitometric analysis of 8 biological replicates of MMP-2 and MMP-9, respectively. ^*∗*^ *p* < 0.05; ^*∗∗*^ *p* < 0.001.

In order to determine the effect of dexamethasone or doxycycline on MMP expression and activation, a gelatin zymogram was performed. To accomplish this, equal amount of protein from each group aortic tissue homogenate was loaded in a 10% PAGE-SDS gel with gelatin as substrate. As shown in Figure 2, under “Control” condition or in the presence of dexamethasone alone, neither MMP-2 nor MMP-9 was detected by gelatin zymogram. In contrast, both MMP-2 and MMP-9 were significantly induced by LPS. Interestingly, however, only MMP-9 but not MMP-2 was activated by LPS in the current study (Figure 2(a)). In the presence of dexamethasone, LPS-induced MMP-9 (both latent and active) and MMP-2 (latent form) were significantly suppressed (Figures 2(b), 2(c), and 2(d), *p* < 0.05). Similarly, doxycycline not only almost completely blocked latent MMP-2 and MMP-9 production, but also significantly inhibited MMP-9 conversion from latent to active form (Figures 2(b), 2(c), and 2(d), *p* < 0.05). Consistent with results of gelatin zymogram, immunoblotting of MMP-2 and MMP-9 confirmed that both dexamethasone and doxycycline could significantly suppress LPS-induced MMP-2 or MMP-9 expression in the aortic tissues (Figures 3(a) and 3(b)).

### 3.4. Effect on ZO-1 Protein Expression

**Figure 4:**
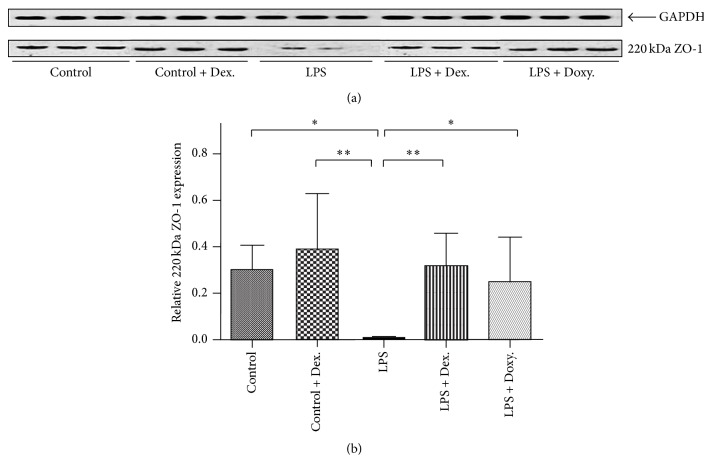
Immunoblotting of ZO-1. Proteins of aortic tissues were extracted from each of the indicated groups and immunoblotted for ZO-1 as described in the method. (a) One representative image data of immunoblotting. GAPDH was used as loading control. Each group was loaded in triplicate. (b) Densitometric analysis data of the relative expression of ZO-1 (mean ± SD, *n* = 8). ^*∗*^ *p* < 0.05; ^*∗∗*^ *p* < 0.001.

Protein levels of ZO-1 in the aortic tissue from each treatment group were examined by immunoblotting. As shown in Figure 4, under “Control” condition or in the presence of dexamethasone alone, ZO-1 was easily detectable in aortic tissue homogenate by immunoblotting assay. When the animals were given LPS, however, ZO-1 expression in the aortic tissue was remarkably decreased (Figures 4(a) and 4(b), *p* < 0.001). Doxycycline could significantly block LPS-induced decline of ZO-1 expression (*p* = 0.006), and, similarly, dexamethasone could also dramatically reduce LPS-induced decline of ZO-1 expression (*p* < 0.05). Although the effect of dexamethasone on ZO-1 restoration in the presence of LPS was less potent than that of doxycycline, there was no statistical difference between these two reagents.

### 3.5. Effect on Syndecan-1 Protein Expression

**Figure 5:**
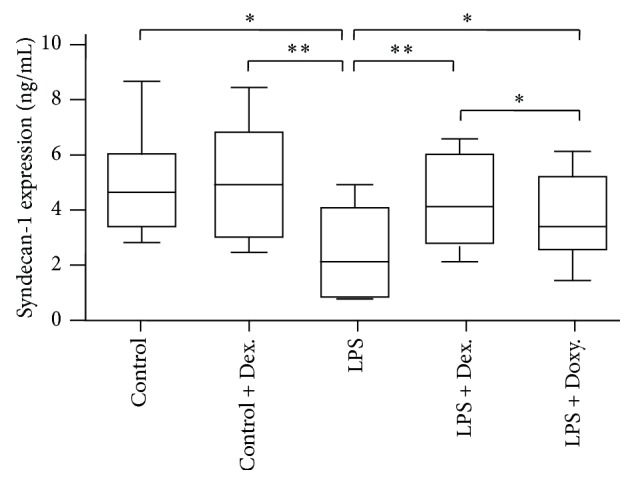
Quantification of syndecan-1 in aortic homogenates by ELISA (mean ± SD, *n* = 8). ^*∗*^ *p* < 0.05; ^*∗∗*^ *p* < 0.001.

In order to further examine the effect of LPS and dexamethasone or doxycycline on endothelial glycocalyx shedding, level of syndecan-1 in the aortic tissue was quantified using a commercially available ELISA assay. As shown in Figure 5, fair amount of syndecan-1 (>4.8 ng/mL) was found in the “Control” condition or in the presence of dexamethasone alone. Consistent with the result of ZO-1 expression, syndecan-1 was significantly suppressed by LPS (2.43 ± 1.56 ng/mL versus 4.93 ± 1.85 ng/mL of Control, *p* < 0.01). Again, both doxycycline and dexamethasone were able to partially but significantly block LPS-induced decline of syndecan-1 expression in the aortic tissues (*p* < 0.05).

## 4. Discussions

Abnormal vascular hyperpermeability is an important feature of infectious shock. Following severe infection or sepsis, inflammatory cytokines accumulate and activate serine proteinases and/or matrix metalloproteinases, which results in increased vascular permeability and loss of barriers of endothelial glycocalyx through shedding the endothelial glycocalyx complex and breaking the cell-cell tight junction [17, 18]. In the current study, we have found that LPS stimulated MMP-2 and MMP-9 production and activation in the rat aortic tissues but significantly decreased expression of ZO-1 and syndecan-1 in the aortic tissues. Both dexamethasone and doxycycline could significantly suppress LPS-induced MMP-2 or MMP-9 production and activation and partially restored LPS-induced suppression of ZO-1 and syndecan-1. Compared to dexamethasone, doxycycline was more potent in suppressing LPS-induced MMP-2 or MMP-9 expression and activation but less potent in restoring ZO-1 or syndecan-1.

Vascular endothelial cells play an important role in maintaining blood-tissue barrier through regulating intracellular and inner-cellular translocation and transportation of large molecules and fluid [8]. Glycocalyx acts on inner-cellular target while tight junctions (TJ) are intracellular molecules, and these structures are important subcellular organs that play role in the pathophysiological process of severe infection or ischemia-reperfusion injury [19]. Syndecans and glypicans are major frame proteins of glycocalyx complex found in almost every endothelial cell layer [10]. ZO-1 is a family of membrane-associated guanylate kinase (MAGUK), localized intracellular cytoplasma of endothelial cells, and connected with other membrane proteins such as occludin and claudins and junctional adhesion molecule (JAM). Combined with TJ protein, ZO-1 not only is a structural protein but also mediates signal transduction between the cells. Therefore, syndecan-1 and ZO-1 were investigated in the current study in order to determine mechanism of glycocalyx shedding in the aortic endothelial cells following severe infection or sepsis.

Matrix metalloproteinases (MMPs) are a family of proteinases containing Zn^2+^-dependent activation domain and released as latent form by a variety of kinds of cells. Latent MMPs can be activated by several ways including protease, acid, and reagents that cause catalytic reaction of conserved glutamate residue and Zn^2+^ ion. Member of MMP family, for instance, MT-MMP1, can also activate other MMPs and leads to a cascade of MMP activation [20]. Once being activated, MMPs degrade extracellular matrixes such as collagen, elastin, and gelatin, and, by which mechanism, MMPs may contribute to the development of septic shock through increasing vascular permeability [21, 22]. In the current study, an endotoxemia model was prepared via intraperitoneal injection of LPS. Dexamethasone or doxycycline was also given to the rat models in order to investigate role of MMPs in modulating aortic endothelial cell permeability. Aortic tissues were harvested and used for assessing MMP-2 and MMP-9 expression and activation by gelatin zymogram and immunoblotting and for assessing glycocalyx associated proteins ZO-1 and syndecan-1. It was found that MMP-2 and MMP-9 were significantly induced and activated by LPS in the rat model thoracic aorta and that both dexamethasone and doxycycline significantly blocked LPS-induction and activation of MMP-9 or induction of MMP-2. Furthermore, LPS caused dramatic reduction of ZO-1 and syndecan-1 expression in the aortic tissues, and both dexamethasone and doxycycline were able to significantly rescue the loss of these two proteins.

Chappell et al. [23, 24] demonstrated that glucocorticoids could improve vascular permeability through protecting the endothelial glycocalyx. Förster et al. [25] and Romero et al. [26] revealed that protection of tight junction proteins could reduce the blood-brain barrier permeability and swelling of brain cell. It is also known that steroid could inhibit MMP expression and activation in a variety of kinds of cells and animal models [14]. Consistent with previous reports, we also found that dexamethasone significantly suppressed MMP-2 expression and MMP-9 expression and activation. In order to examine the role of MMP in glycocalyx shedding, a pan MMP inhibitor, doxycycline was also used in parallel with dexamethasone. Since MMPs are not produced under normal physiological condition, doxycycline alone control was not included in the current study. We demonstrated that dexamethasone and doxycycline had similar biological effect not only on MMP-2 and MMP-9, but also on the expression of ZO-1 and syndecan-1 in response to LPS injection. In this context, LPS-induced MMP-2 and MMP-9 were significantly suppressed while ZO-1 and syndecan-1 were significantly restored by dexamethasone and doxycycline, suggesting dexamethasone and doxycycline may prevent glycocalyx from shedding by suppressing MMP-2 and MMP-9.

The limitation of the current study is lack of mechanistic investigation on direct or indirect biological effect of dexamethasone on MMP activation and production, especially when considered in comparison to the more direct inhibitory effects of doxycycline on MMPs.

Taken together, LPS induced significant upregulation and activation of MMP-2 and MMP-9 in the rat endotoxemia model, which may contribute to the development of vascular leaking through shedding endothelial surface glycocalyx complex. Dexamethasone and doxycycline may protect endothelial cells from injury and glycocalyx from shedding through suppressing MMP-2 and MMP-9 in LPS-induced rat endotoxemia model. Detailed mechanism behind this phenomenon remains to be further defined.

## Supplementary Material

The profile of animal modeling process.LPS (10mg/Kg) was intraperitonealy injected. Dexamethasone (4 mg/kg) was injected 30 minutes before LPS injection in the groups of dexamethasone alone or LPS+dexamethasone. Doxycycline (4 mg/kg) was injected 30 minutes after LPS injection. All animals were sacrificed humanely by pentobarbital sodium overdose (100 mg/kg ip) after 6 hours of LPS injection.

## Figures and Tables

**Table 1:** Comparison of blood gas analysis (mean ± SD).

Variables	Control	Dex.	LPS	LPS + Dex.	LPS + Doxy.
Lactate, mmol/L	1.9 ± 0.3^**∗**^	2.3 ± 0.4	6.1 ± 3.7	2.3 ± 0.7^**∗**^	2.7 ± 0.9^**∗**^
pH	7.36 ± 0.03^**∗∗**^	7.38 ± 0.07^**∗∗**^	7.18 ± 0.12	7.39 ± 0.04^**∗∗**^	7.36 ± 0.06^**∗∗**^
BE, mmol/L	5.3 ± 2.9^**∗**^	6.3 ± 0.9^**∗**^	2.4 ± 7.1	4.4 ± 1.2^**∗**^	4.3 ± 1.0^**∗**^
PaCO_2_, mmHg	57.9 ± 10.8	57.1 ± 13.5^**∗**^	66.8 ± 4.4	50.6 ± 5.4^**∗∗∗**^	57.6 ± 10.2^**∗**^
SaO_2_, %	87.8 ± 10.1^**∗**^	88.3 ± 9.2	77.9 ± 4.3	94.0 ± 3.4^**∗∗∗**^	86.0 ± 8.7^**∗**^

Compared with group endotoxin, ^**∗**^ *p* < 0.05; ^**∗∗**^ *p* < 0.01; ^**∗∗∗**^ *p* < 0.001.

BE, base excess; PaCO_2_, arterial carbon dioxide pressure, SaO_2_, arterial oxygen saturation.
