# Supplementary figures and images for: Dexamethasone Suppressed LPS-Induced Matrix Metalloproteinase and Its Effect on Endothelial Glycocalyx Shedding
Source: Mediators Inflamm. 2015 Jun 23;2015:912726. doi: 10.1155/2015/912726 (PMC4493300; doi:10.1155/2015/912726)

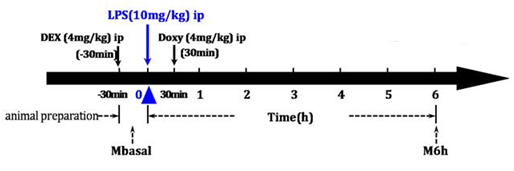

Supplement: Supplementary file 1 — The profile of animal modeling process.LPS (10mg/Kg) was intraperitonealy injected. Dexamethasone (4 mg/kg) was injected 30 minutes before LPS injection in the groups of dexamethasone alone or LPS+dexamethasone. Doxycycline (4 mg/kg) was injected 30 minutes after LPS injection. All animals were sacrificed humanely by pentobarbital sodium overdose (100 mg/kg ip) after 6 hours of LPS injection. [file 912726.f1.tif]
